# Ethyl *p*-methoxycinnamate inhibits tumor growth by suppressing of fatty acid synthesis and depleting ATP

**DOI:** 10.1038/s41598-025-00131-1

**Published:** 2025-05-02

**Authors:** Yutaro Sasaki, Niina Mizushima, Toshio Norikura, Isao Matsui-Yuasa, Akiko Kojima-Yuasa

**Affiliations:** 1 Department of Nutrition, Graduate School of Human Life and Ecology, Osaka Metropolitan University, Osaka, 558-8585 Japan; 2 Department of Nutrition, Aomori University of Health and Welfare, Aomori, 030-8505 Japan

**Keywords:** *Kaempferia galanga* L., Ethyl *p*-methoxycinnamate, *De Novo* fatty acid synthesis, Glycolysis, ATP, Ehrlich Ascites tumor cells, Cancer, Cancer metabolism

## Abstract

Cancer cells reprogram their energy metabolism pathways, but the mechanisms that enable them to meet their energy demands remain poorly understood. This study investigates the anticancer effects of ethyl *p*-methoxycinnamate (EMC) in Ehrlich ascites tumor cells (EATCs) and reveals that *de novo* fatty acid synthesis, rather than glycolysis, plays a pivotal role in sustaining energy homeostasis in cancer cells. EMC significantly reduced ATP levels despite enhancing glycolytic activity. It suppressed the expression of key enzymes involved in *de novo* fatty acid synthesis, including *Acly*, *Acc1*, and *Fasn*, resulting in decreased intracellular triglyceride (TG) levels. The addition of exogenous palmitic acid reversed EMC-induced ATP depletion and mitigated its anti-proliferative effects. Mechanistically, the ATP reduction caused by EMC was associated with inhibition of the c-Myc/SREBP1 pathway and arrest of the G1/S cell cycle transition. These findings demonstrate that EMC inhibits EATC proliferation by reducing ATP levels via suppression of *de novo* fatty acid synthesis. This study highlights the critical role of *de novo* fatty acid synthesis, rather than glycolysis, in maintaining energy homeostasis in cancer cells and provides novel insights into targeting cancer metabolism.

**Supplementary Information:**

The online version contains supplementary material available at 10.1038/s41598-025-00131-1.

## Introduction

Cancer cells require substantial amounts of ATP to sustain their rapid proliferation and survival. When intracellular energy deficits are sensed at cell cycle checkpoints, cells may arrest the cell cycle or exit it altogether to prevent uncontrolled division and potential cell damage^1^. To meet the increased energy demands, cancer cells reprogram their metabolic pathways for glucose, fatty acids, and amino acids^2^. A fundamental feature of cancer metabolism is the Warburg effect, a phenomenon where cancer cells predominantly generate ATP through glycolysis rather than oxidative phosphorylation, even in oxygen-rich conditions. Initially observed by Warburg in Ehrlich ascites tumor cells (EATCs), this effect has since been demonstrated across various cancer cell types^3–5^. Based on the Warburg effect, it has been suggested that cancer cells rely on glycolysis for ATP production due to impaired mitochondrial function^6,7^.

However, recent studies have questioned this assumption, showing that mitochondria in cancer cells are not necessarily impaired and retain various functional capacities, including oxidative phosphorylation, even in highly glycolytic cancer cells^8^. Furthermore, recent research has reported that fatty acid metabolism plays an important role as the primary ATP source rather than glycolysis in some cancer cells^9^. Both *de novo* fatty acid synthesis and fatty acid oxidation are increasingly recognized as prominent features of cancer cell metabolism^10^. To meet fatty acid demand, dietary fatty acids are utilized in normal cells, but *de novo* fatty acid synthesis is preferred in cancer cells^11,12^. These findings underscore the complexity of cancer cell energy metabolism, as it involves coordinated reprogramming of multiple metabolic pathways, making a comprehensive understanding of cancer metabolism challenging and subject to ongoing research.

Natural compounds have garnered significant interest in cancer prevention for several decades due to their potent anticancer effects and low toxicity^13^. Compounds such as curcumin, resveratrol, and epigallocatechin gallate have been shown to inhibit the proliferation of cancers, including colorectal, liver, nasopharyngeal, breast, and pheochromocytoma, by modulating energy metabolism^14,15^. However, most studies have focused on the effects of these compounds on specific metabolic pathways, often without examining the broader interactions within energy metabolism. *Kaempferia galanga* L., a medicinal plant from the Zingiberaceae family, contains ethyl *p*-methoxycinnamate (EMC) as a primary active compound^16,17^. Our recent studies have demonstrated that *Kaempferia galanga* L. extract and EMC inhibit the proliferation of EATCs by suppressing the G1 to S phase transition through a reduction in the transcriptional activity of the oncogene c-Myc^18^. However, the effects of EMC on energy metabolism in EATCs remain unclear. Therefore, this study aimed to elucidate the anticancer mechanisms of EMC by investigating its influence on energy metabolism, focusing on glycolysis and fatty acid metabolism. This comprehensive investigation seeks to provide a deeper understanding of the interplay between metabolic pathways in cancer cells, using EATCs as a model to evaluate the impact of EMC on energy metabolism.

## Results

### Effect of EMC on Glycolysis and ATP levels in EATCs

**Fig. 1:**
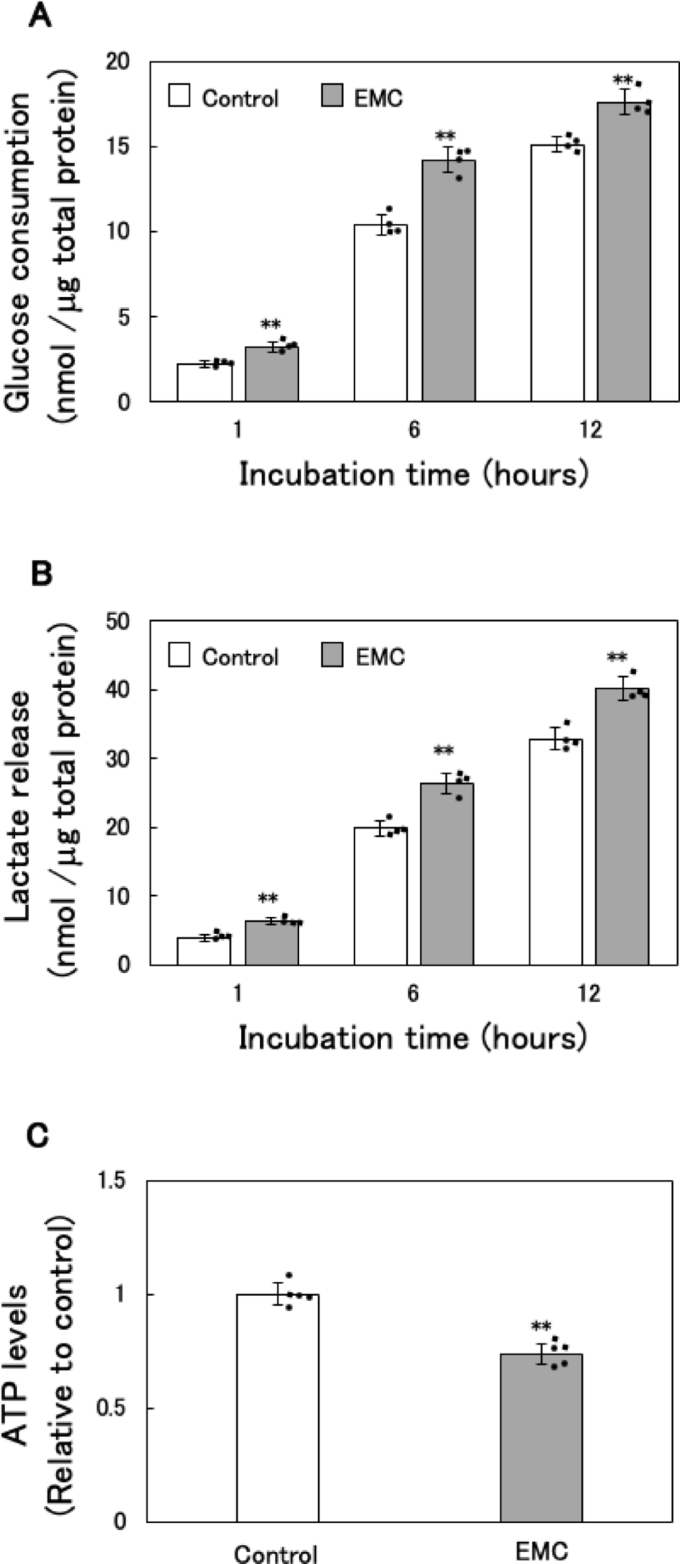
Effect of EMC on glycolysis and ATP levels in EATCs. EATCs were treated with or without 100 µM EMC for various time points. (A) The glucose consumption levels in the medium were determined by the Glucose Assay Kit-WST. Results are expressed as mean ± SD (*n* = 4). (B) The lactate release levels in the medium were determined by the Lactate Assay Kit-WST. Results are expressed as mean ± SD (*n* = 4). (C) ATP levels were determined by the CellTiter-Glo 2.0 Assay. Results are expressed as mean ± SD (*n* = 5). Statistical significance between the two groups was evaluated using Student’s t-test. The data were found to be significantly different compared to the control: ** *p* < 0.01.

In a previous study, we demonstrated that 100 µM EMC inhibits the proliferation of EATCs without cytotoxic effects on normal cells^18^. Based on these findings, a concentration of 100 µM EMC was employed in the present study. Considering the Warburg effect, EMC’s anticancer effect in EATCs may involve ATP depletion through glycolysis inhibition. To evaluate this hypothesis, we examined glucose consumption and lactate production in the culture medium of EMC-treated EATCs. The same medium samples were analyzed for both glucose consumption and lactate release. EATCs treated with EMC significantly increased glucose consumption in the culture medium (Fig. 1A). Lactate release levels were also significantly increased in EMC-treated EATCs (Fig. 1B). Conversely, intracellular ATP levels were significantly decreased following EMC treatment (Fig. 1C).

### Effect of EMC on fatty acid synthesis and oxidation in EATCs

**Fig. 2:**
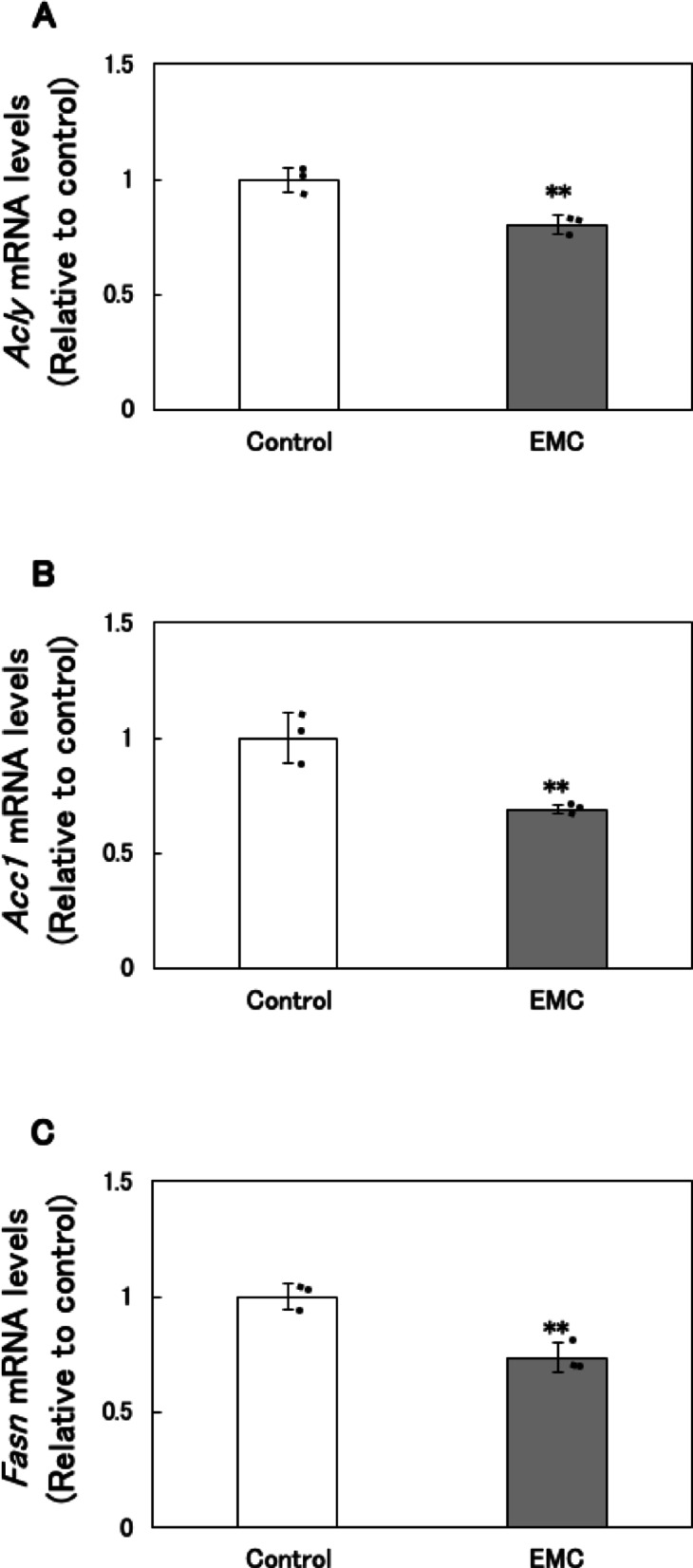

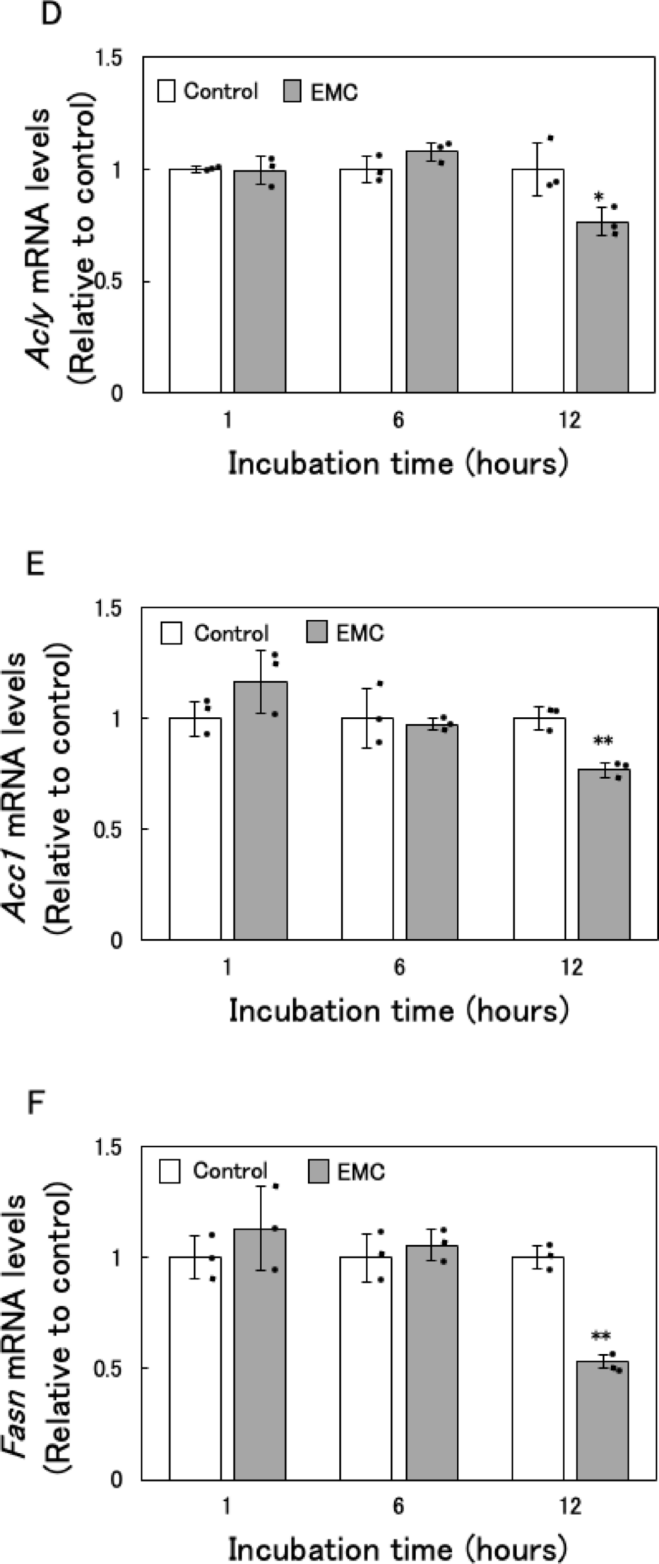

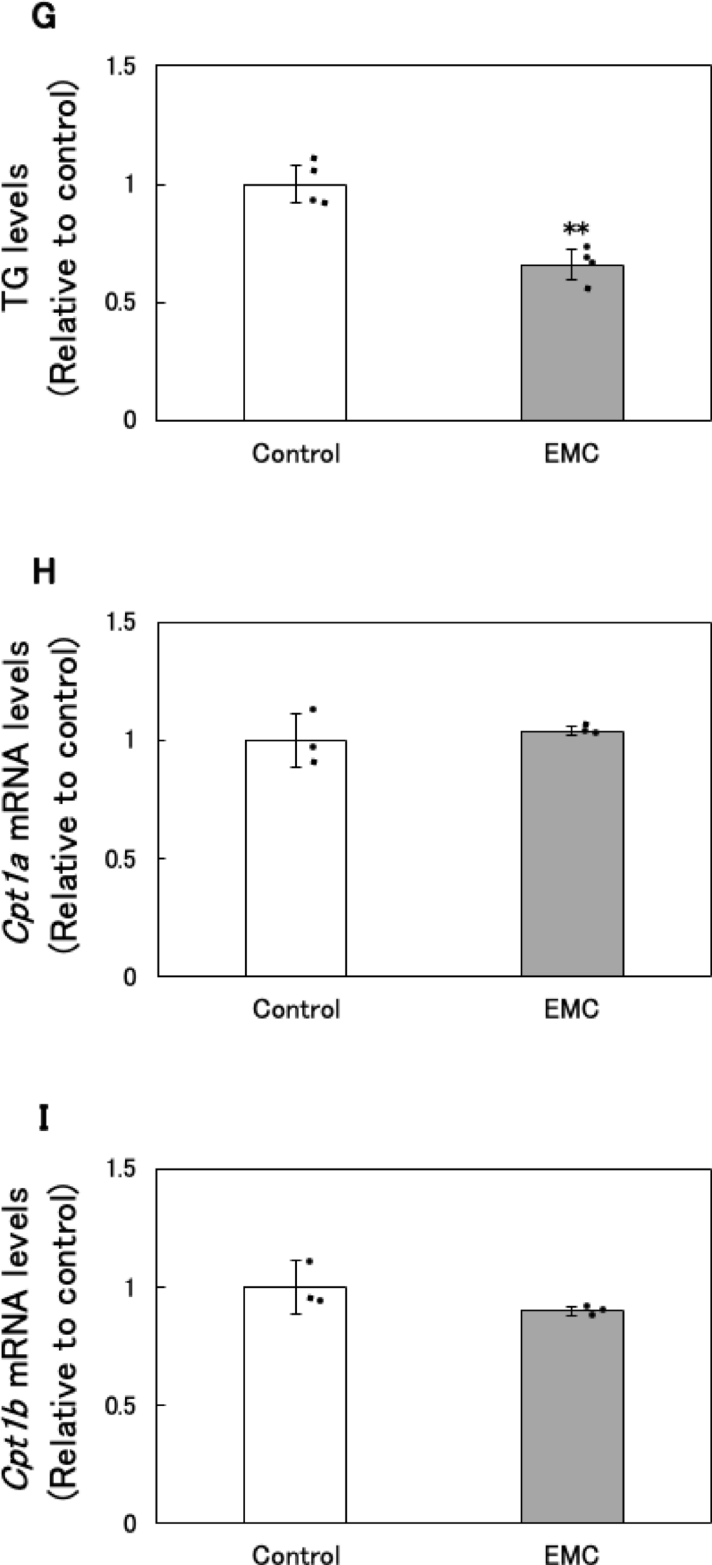
Effect of EMC on fatty acid synthesis and oxidation in EATCs. (A-C, H, I) EATCs were treated with or without 100 µM EMC for 12 h. (D-F) EATCs were treated with or without 100 µM EMC for various time points. mRNA levels of genes related to *de novo* fatty acid synthesis (*Acly*, *Acc1*, and *Fasn*) and oxidation (*Cpt1a* and *Cpt1b*) determined by using qRT-PCR. Results are expressed as mean ± SD (*n* = 3). (G) Intracellular TG levels were determined by LabAssay Triglyceride. Results are expressed as mean ± SD (*n* = 4). Statistical significance between the two groups was evaluated using Student’s t-test. The data were found to be significantly different compared to the control: ** *p* < 0.01.

To investigate the mechanism by which EMC reduces ATP levels, we examined its effects on fatty acid synthesis and oxidation. Key genes involved in *de novo* fatty acid synthesis, including ATP citrate lyase (*Acly*), acetyl-CoA carboxylase alpha (*Acc1*), and fatty acid synthase (*Fasn*), which collectively convert citrate to PA, were analyzed. The mRNA expression levels of *Acly*, *Acc1*, and *Fasn* were significantly downregulated in EMC-treated EATCs 12 h after treatment (Fig. 2A, B and C). However, the mRNA levels of these fatty acid synthesis-related genes did not significantly change at 1–6 h after EMC treatment (Fig. 2D, E and F).

*De novo* synthesized fatty acids are typically incorporated into triglycerides (TGs), a primary component of lipid droplets used for energy storage in cancer cells^19^. We therefore measured intracellular TG levels and found a significant decrease in TG content in EMC-treated EATCs (Fig. 2G).

To assess the effect of EMC on fatty acid oxidation, we measured mRNA expression levels of *carnitine palmitoyltransferase 1a* (*Cpt1a*) and *carnitine palmitoyltransferase 1b* (*Cpt1b*), enzymes essential for fatty acid oxidation. No significant changes were observed in *Cpt1a* and *Cpt1b* mRNA expression levels following EMC treatment (Fig. 2H and I).

### Effect of co-addition of EMC and palmitic acid (PA) on ATP levels in EATCs

**Fig. 3:**
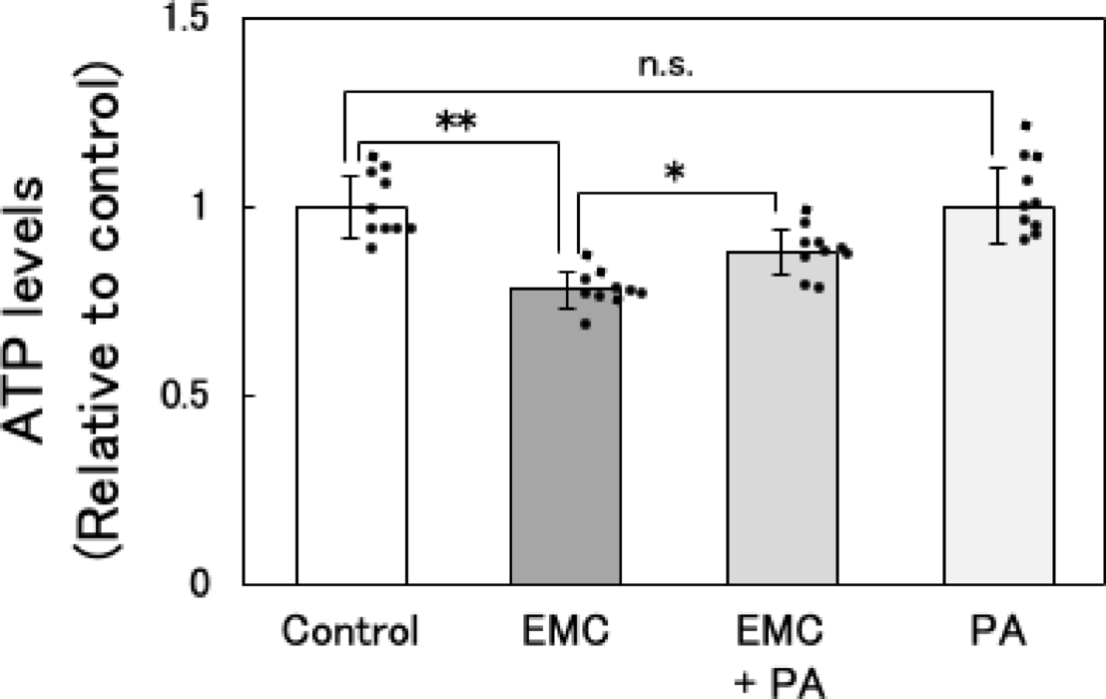
Effect of co-addition of EMC and PA on ATP levels in EATCs. EATCs were treated with 100 µM EMC, 40 µM palmitic acid (PA), a combination of both, or left untreated for 12 h. ATP levels were determined by the CellTiter-Glo 2.0 Assay. Results are expressed as mean ± SD (*n* = 10). Statistical significance between the four groups was evaluated using one-way ANOVA, followed by the Tukey-Kramer test. The data were found to be significantly different: * *p* < 0.05 and ** *p* < 0.01.

To determine if the reduction in ATP levels caused by EMC is related to its inhibition of *de novo* fatty acid synthesis, exogenous PA was added to the culture medium to restore the intracellular fatty acid levels decreased by the inhibition of *de novo* synthesis, and ATP levels were subsequently measured. EMC treatment significantly decreased ATP levels; however, this reduction was effectively reversed by the addition of exogenous PA (Fig. 3). There was no significant difference in ATP levels between the control group and the palmitic acid alone group.

### Effect of EMC on ATP levels and DNA content in EATCs

**Fig. 4:**
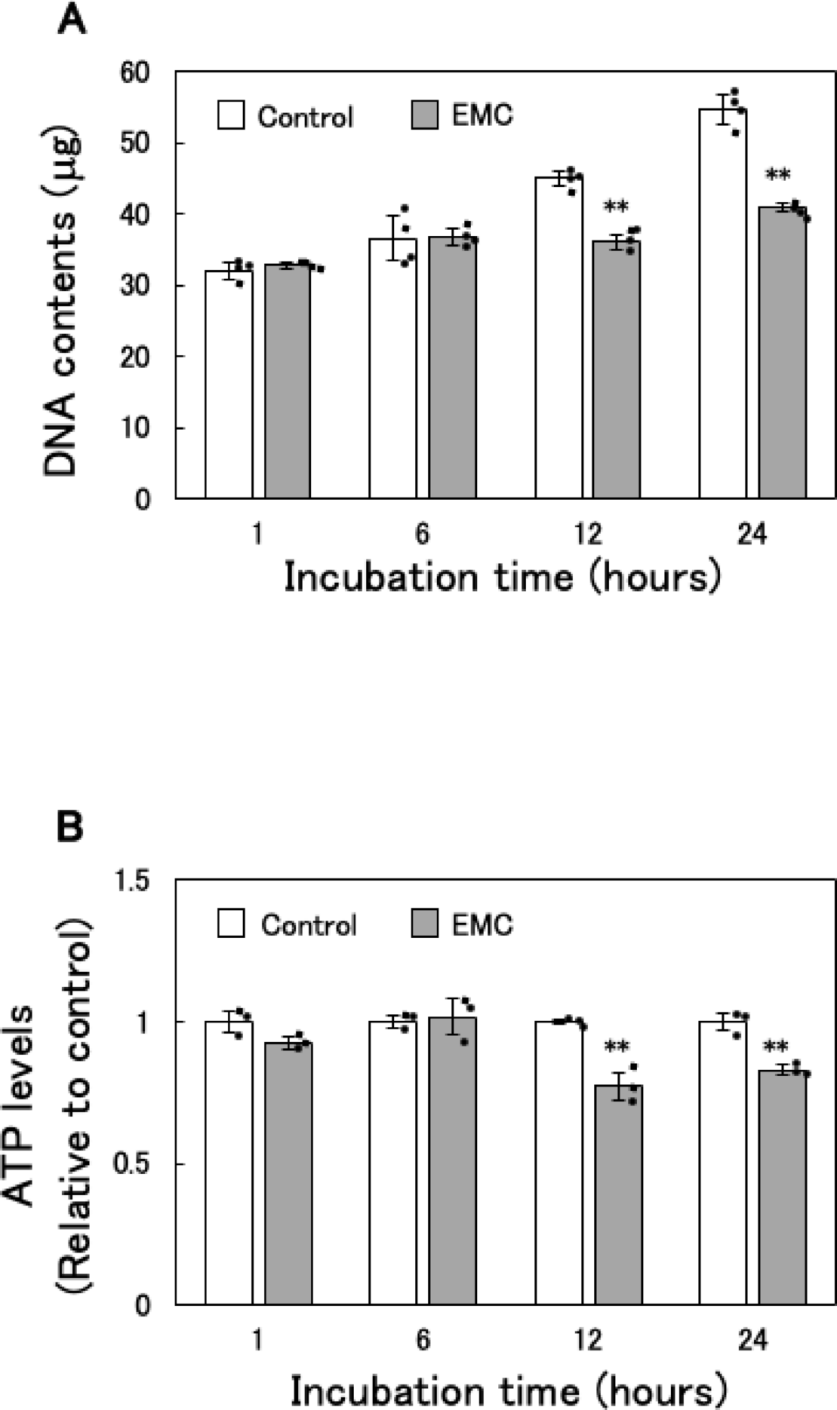
Effect of EMC on ATP levels and DNA content in EATCs. EATCs were treated with or without 100 µM EMC for various times. (A) DNA content was determined using diphenylamine. Results are expressed as mean ± SD (*n* = 4). (B) ATP levels were determined by the CellTiter-Glo 2.0 Assay. Results are expressed as mean ± SD (*n* = 3). Statistical significance between the two groups was evaluated using Student’s t-test. The data were found to be significantly different comparison to the control: ** *p* < 0.01.

Cellular energy shortage is detected by cell cycle checkpoints, which can result in cell cycle arrest^1^. We previously demonstrated that EMC inhibits the G1 to S phase transition in EATCs^18^. Based on this, we examined the relationship between EMC-induced ATP reduction and the inhibition of G1/S phase transition. An increase in DNA content over time indicates that EATCs have progressed from the G1 to S phase. EATCs were treated with or without EMC and analyzed at 1, 6, 12, and 24 h. Up to 6 h after EMC treatment, there was no significant difference in DNA content between the groups (Fig. 4A). However, at time points beyond 12 h, the control group exhibited an increase in DNA content, whereas this increase was suppressed in the EMC-treated EATCs. Similarly, ATP levels showed no significant difference up to 6 h after EMC treatment but were significantly reduced in the EMC-treated group after 12 h (Fig. 4B). These results indicate that EMC-induced ATP depletion coincides with the inhibition of the G1 to S phase transition.

### Effect of co-addition of EMC and PA on cell number and viability of EATCs

**Fig. 5:**
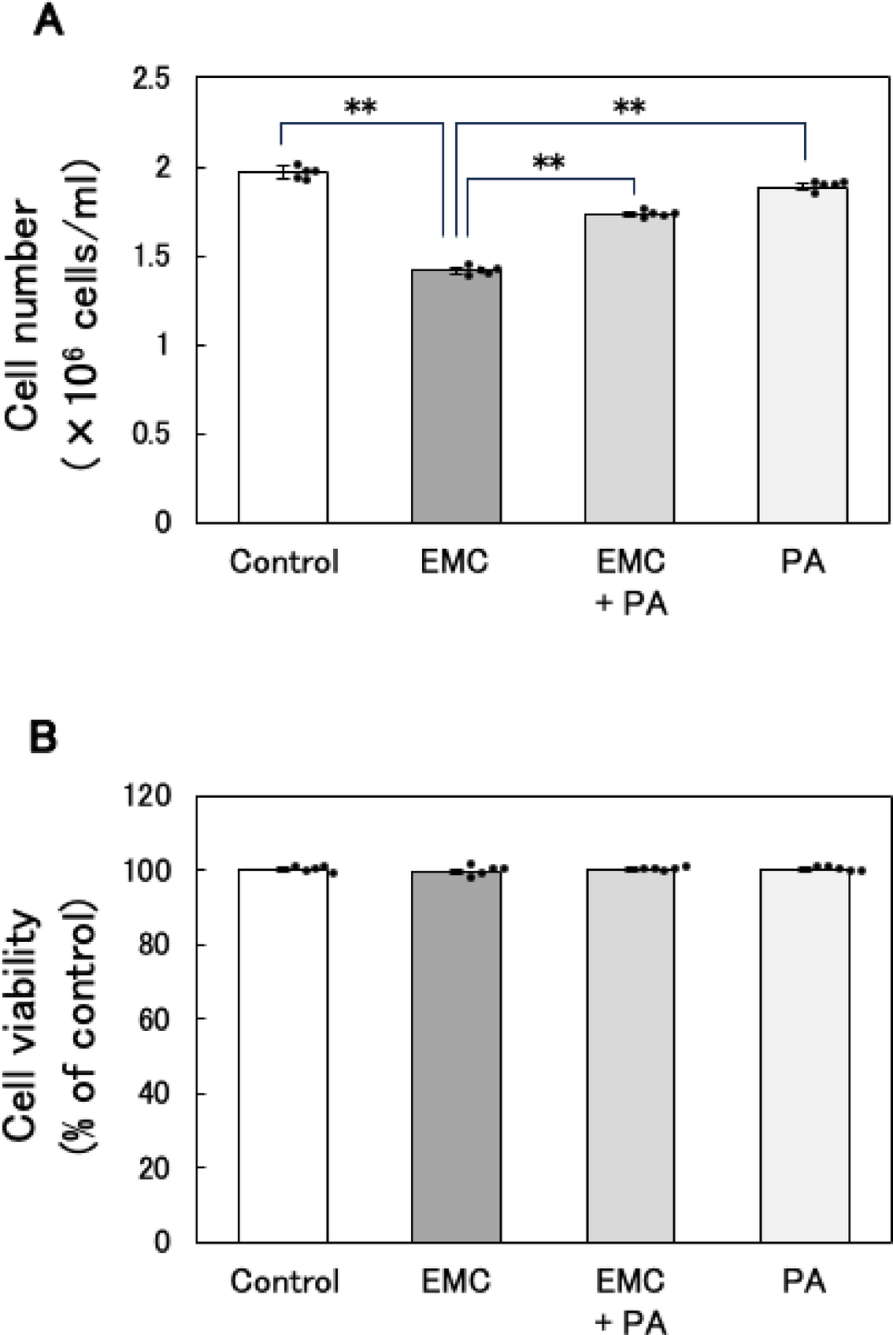
Effect of co-addition of EMC and PA on cell number and viability of EATCs. EATCs were treated with 100 µM EMC, 40 µM palmitic acid (PA), a combination of both, or left untreated for 24 h. (A) Cell number and (B) cell viability were determined using trypan blue. Results are expressed as mean ± SD (*n* = 5). Statistical significance between the four groups was evaluated using one-way ANOVA, followed by the Tukey-Kramer test. The data were found to be significantly different comparison to the control: ** *p* < 0.01.

To investigate whether the anti-proliferative effect of EMC on cancer cells is related to intracellular fatty acid depletion, we supplemented the culture medium with exogenous PA and evaluated its effect on cell number and viability in EATCs. EMC treatment significantly reduced cell number; however, this reduction was reversed by the addition of exogenous PA (Fig. 5A). This result indicate that fatty acid depletion is a contributing factor in EMC-induced suppression of EATC proliferation. In contrast, no significant differences in cell viability were observed across the treatment groups, indicating that apoptosis was not induced in any of the groups (Fig. 5B).

### Effect of EMC on sterol regulatory element binding transcription factor 1 (SREBP1) expression levels in EATCs

**Fig. 6:**
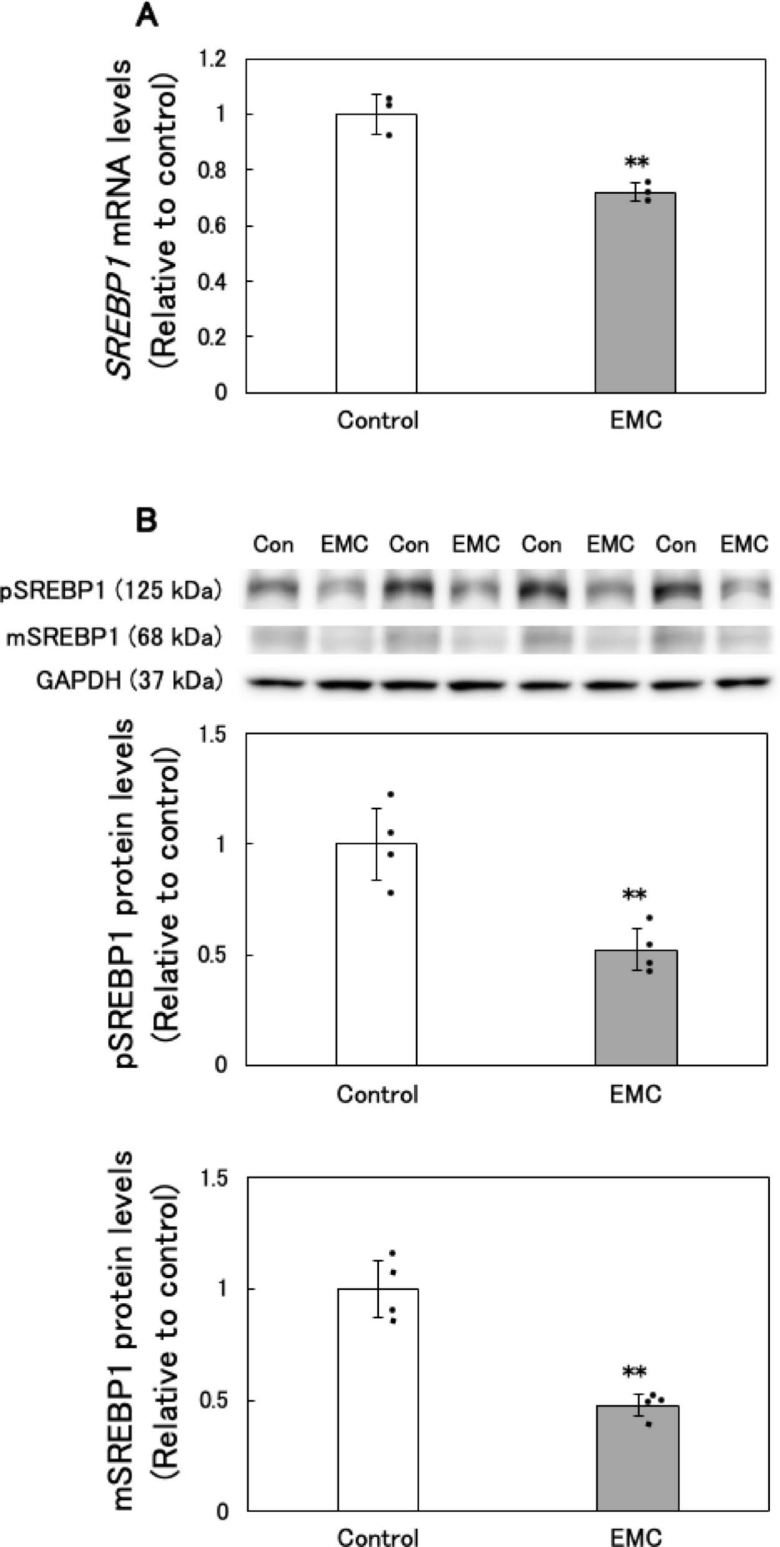
Effect of EMC on sterol regulatory element binding transcription factor 1 (SREBP1) expression levels in EATCs. EATCs were treated with or without 100 µM EMC for 12 h. (A) SREBP1 mRNA expression levels were determined by qRT-PCR. Results are expressed as mean ± SD (*n* = 3). (B) SREBP1 protein expression levels were determined by Western blot analysis. The images of pSREBP1 and mSREBP1 were obtained from the same blot with different exposure times. After detecting SREBP1, the membrane was stripped of the SREBP1 antibody and reprobed with GAPDH antibody to obtain the GAPDH bands. Results are expressed as mean ± SD (*n* = 4). Statistical significance between the two groups was evaluated using Student’s t-test. The data were found to be significantly different comparison to the control: ** *p* < 0.01.

To further elucidate the mechanism behind EMC-induced inhibition of *de novo* fatty acid synthesis, we examined the expression of SREBP1, a key regulator of genes involved in fatty acid and triglyceride biosynthesis^20^. SREBP1 directly regulates the expression of target genes including *Acly*, *Acc1*, and *Fasn*^21^. Our results showed that EMC treatment significantly reduced *SREBP1* mRNA levels in EATCs (Fig. 6A).

SREBP1 protein is synthesized as a precursor (pSREBP1) and initially resides in the endoplasmic reticulum. Upon transport to the Golgi apparatus, pSREBP1 undergoes cleavage by Site-1 and Site-2 proteases to generate the mature, active form (mSREBP1), which then translocates to the nucleus to function as a transcription factor^22^. As shown in Fig. 6B, both pSREBP1 and mSREBP1 protein levels were significantly decreased in EMC-treated EATCs.

### Effect of EMC on the levels of phosphorylated c-Myc at Serine 62 in EATCs

**Fig. 7:**
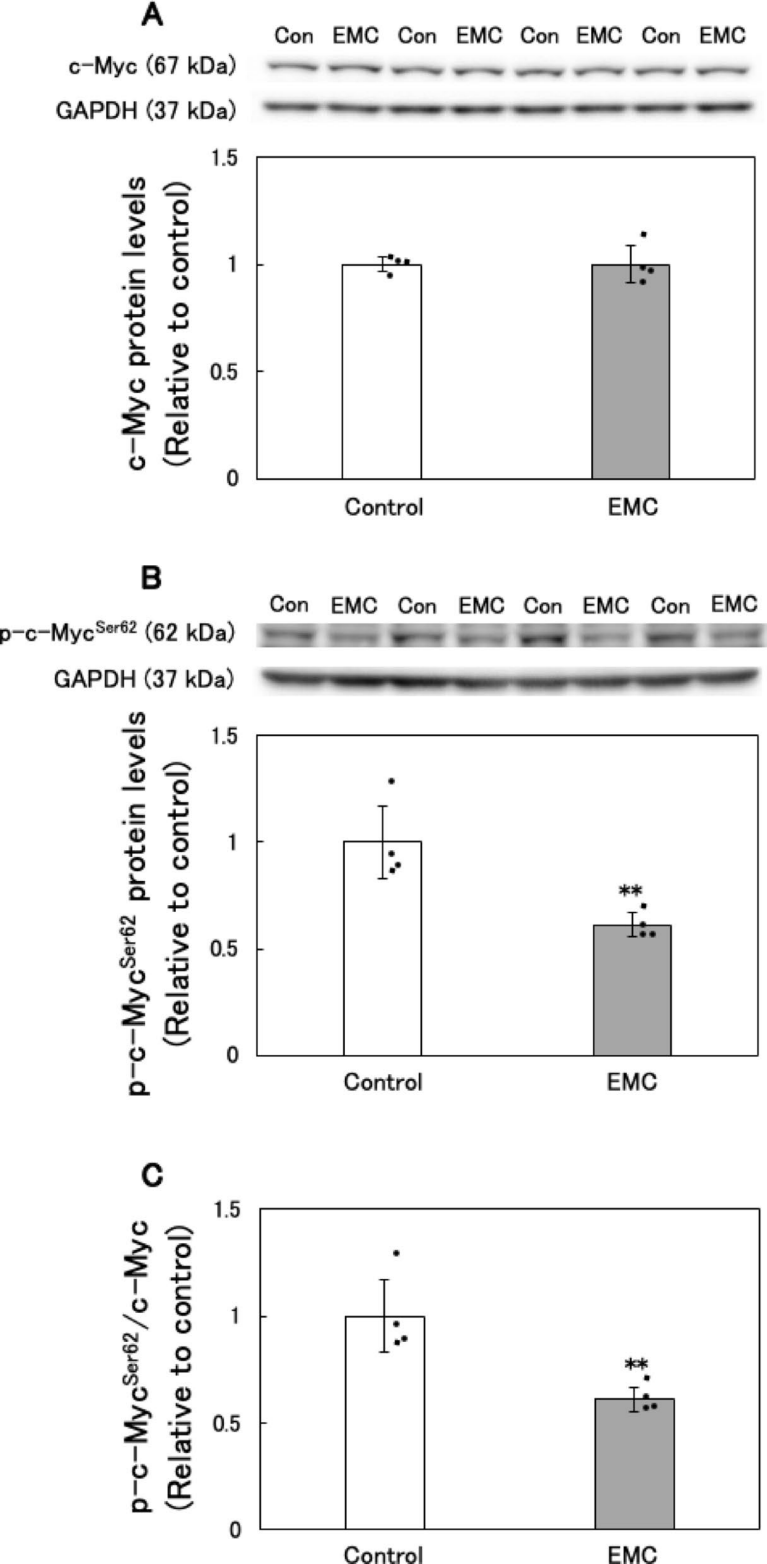
Effect of EMC on the levels of phosphorylated c-Myc at Ser-62 in EATCs. EATCs were treated with or without 100 µM EMC for 6 h. The levels of (A) c-Myc and (B) phosphorylated c-Myc at Ser-62 were determined by Western blot analysis. The images of c-Myc and GAPDH were obtained from the same blot with different exposure times. After detecting c-Myc, the membrane was stripped of the c-Myc antibody and reprobed with GAPDH antibody to obtain the GAPDH bands. The images of the phosphorylated c-Myc at Ser-62 and GAPDH were obtained from the same blot but with different exposure times. (C) Results show the ratio of phosphorylated c-Myc at Ser-62 to c-Myc. Results are expressed as mean ± SD (*n* = 4). Statistical significance between the two groups was evaluated using Student’s t-test. The data were found to be significantly different comparison to the control: ** *p* < 0.01.

The oncogene c-Myc directly binds to the promoter region of SREBP1, thereby activating its transcriptional expression^23^. Phosphorylation of Ser62 on c-Myc enhances its transcriptional activity^24^. In a previous study, we showed that EMC treatment suppresses phosphorylation at serine 62 of c-Myc in EATCs after 12 h^18^. In this study, we examined the effect of EMC on c-Myc phosphorylation at serine 62 at an earlier time point to gain further insights into the mechanisms underlying EMC’s anticancer effects via c-Myc regulation. As shown in Fig. 7A and B, EMC did not affect the total expression levels of c-Myc but significantly reduced the levels of serine 62-phosphorylated c-Myc. These results indicated that EMC significantly decreased the levels of serine 62-phosphorylated c-Myc compared to the total c-Myc expression levels (Fig. 7C).

## Discussion

Cancer cells are characterized by their ability to reprogram energy metabolism to sustain uncontrolled proliferation, leading to distinct metabolic traits compared to normal cells^25^. Despite significant advances in understanding ATP-generating pathways in cancer biology, the mechanisms underlying ATP reduction in cancer cells remain poorly understood. In this study, we provide a novel perspective on cancer metabolism by demonstrating that EMC suppresses ATP production via the inhibition of *de novo* fatty acid synthesis, which is accompanied by enhanced glycolytic activity. Notably, the ATP reduction was significantly reversed by exogenous supplementation with PA, highlighting the critical role of *de novo* fatty acid synthesis in maintaining energy homeostasis in cancer cells. These findings suggest that targeting *de novo* fatty acid synthesis could be a promising therapeutic strategy for cancer treatment.

ATP is produced from carbon substrates via two distinct metabolic pathways: glycolysis and mitochondrial oxidative phosphorylation. Glycolysis has long been regarded as the primary ATP-generating pathway in cancer cells^7^. However, our results revealed a paradoxical increase in glycolytic activity, evidenced by enhanced glucose uptake and lactate production, despite EMC-induced ATP depletion. This finding is particularly significant, as it indicates that ATP reduction is not caused by glycolytic inhibition. While mitochondrial dysfunction has been implicated in various metabolic shifts in cancer cells, emerging evidence, including our study, challenges this assumption. Specifically, synthetic phosphoethanolamine has been shown to induce apoptosis via mitochondrial membrane potential loss in EATCs^26^, reinforcing the importance of mitochondria as a therapeutic target. Taken together, our findings emphasize the complementary roles of glycolysis and mitochondrial oxidative phosphorylation in sustaining the high ATP demands of cancer cells.

A key finding of this study is that EMC-induced ATP depletion primarily results from the inhibition of *de novo* fatty acid synthesis. Our previous study has demonstrated that EMC did not affect the mitochondrial membrane potential and mitochondrial DNA copy number in EATCs^18^, suggesting that there is no inhibition of mitochondrial ATP synthesis activity. This finding further supports the idea that EMC-induced ATP depletion is unrelated to mitochondrial dysfunction, suggesting the involvement of other energy production processes. Fatty acids serve as one of the energy substrates for ATP production in mitochondria. There are distinct differences in fatty acid metabolism between normal and cancer cells. While normal cells utilize exogenous fatty acids, cancer cells prefer endogenous fatty acids to sustain their uncontrolled proliferation^12,27^. In human cancer tissues, *Acly*, *Acc1*, and *Fasn* are overexpressed compared to normal tissues^28^, and *de novo* synthesized fatty acids are efficiently oxidized and utilized as substrates for ATP production in cancer cells^29,30^. In this study, EMC did not significantly affect the expression levels of *Cpt1a* and *Cpt1b*, the rate-limiting enzymes of fatty acid oxidation. However, EMC significantly reduced the expression of key enzymes involved in *de novo* fatty acid synthesis, including *Acly*, *Acc1*, and *Fasn*. Furthermore, intracellular triglyceride levels were also reduced. The restoration of ATP levels by PA supplementation further supports the notion that *de novo* fatty acid synthesis is essential for ATP production in cancer cells. Additionally, the expression levels of *Acly*, *Acc1*, and *Fasn* remained unchanged at 1 and 6 h after EMC treatment but were significantly downregulated at 12 h. There was no temporal relationship between the expression of these fatty acid synthesis-related genes and glucose consumption. Therefore, it was suggested that fluctuations in glucose consumption are not involved in the downregulation of fatty acid synthesis-related gene expression. This mechanistic insight highlights the unique vulnerability of cancer cells to disruptions in fatty acid metabolism.

Cancer cells are known for their ability to adapt to energy stress by reprogramming metabolic pathways. For example, inhibition of mitochondrial oxidative phosphorylation often leads to enhanced glycolysis^31^. Similarly, our study demonstrates for the first time that EMC-induced inhibition of *de novo* fatty acid synthesis triggers a compensatory upregulation of glycolysis, potentially as a survival mechanism. This adaptive response likely contributes to cancer cell survival under metabolic stress and is recognized as a key mechanism underlying resistance to metabolism-targeting therapies^32^. While EMC inhibits EATC proliferation, its failure to induce cell death may be attributed to this metabolic plasticity. These findings underscore the potential of combining *de novo* fatty acid synthesis and glycolysis inhibitors as a more effective strategy for suppressing cancer cell growth.

This study also provides important evidence linking ATP depletion to cell cycle arrest at the G1/S phase transition. Previous reports indicate that ATP levels peak during the transition from G1 to S phase and that compounds such as gramicidin A inhibit this transition by depleting ATP^33,34^. Our results show that EMC-induced ATP reduction coincides with G1/S phase arrest and that the suppression of EATC proliferation by EMC is reversed by PA supplementation. These findings suggest that ATP depletion caused by the inhibition of *de novo* fatty acid synthesis disrupts energy-dependent cell cycle progression, thereby suppressing EATC proliferation. This mechanistic link between fatty acid metabolism, ATP production, and cell cycle regulation highlights a critical vulnerability that can be exploited for therapeutic purposes.

 Schcolnik-Cabrera et al. have shown that orlistat is an appropriate positive control for FASN inhibition and demonstrated that orlistat induces apoptosis in colon cancer cells by regulating fatty acid metabolism^35,36^. In contrast, our results showed that EMC suppressed cancer cell proliferation without inducing apoptosis. Given this difference and the absence of a suitable positive control that aligns with the mechanism of action of EMC, we were unable to include a FASN inhibitor as a positive control in this study. In future studies, establishing an appropriate positive control will be crucial for further elucidating the specific effects of EMC.

Another novel finding of this study is the identification of SREBP1 and c-Myc as key regulators of EMC-induced inhibition of *de novo* fatty acid synthesis. SREBP1 serves as a master regulator of *de novo* fatty acid biosynthesis, while c-Myc functions as its transcriptional regulator^20,23^. SREBP1 was significantly downregulated at both the mRNA and protein levels following EMC treatment. Additionally, EMC inhibited c-Myc phosphorylation at Ser62. Inhibition of Ser62 phosphorylation reduces c-Myc’s binding affinity for the promoter regions of target genes, leading to a decrease in transcriptional activity^37,38^. These findings reveal a mechanism by which EMC disrupts fatty acid metabolism through the inactivation of the c-Myc/SREBP1 axis. Further studies are required to elucidate how EMC inhibits c-Myc phosphorylation and to explore its broader implications in cancer biology.

In conclusion, this study demonstrates that EMC suppresses EATC proliferation by reducing ATP levels through the inhibition of *de novo* fatty acid synthesis, rather than glycolysis. Furthermore, this ATP depletion is linked to the inactivation of the c-Myc/SREBP1 pathway and disruption of the G1/S phase transition. These findings provide critical new insights into the metabolic vulnerabilities of cancer cells and underscore the therapeutic potential of targeting *de novo* fatty acid synthesis in combination with glycolysis inhibition. To fully understand the impact of EMC on cancer cell metabolism, future studies should explore its effects on other pathways, such as glutamine metabolism, the pentose phosphate pathway, and the TCA cycle.

## Materials and methods

### Cell culture

EATCs (JCRB9090), spontaneously derived cancer cells collected from mouse ascites fluid, were obtained from the Japanese Cancer Research Resource Bank (Tokyo, Japan). EATCs were grown in Dulbecco’s modified Eagle’s medium (DMEM; 05915, Nissui Pharmaceutical, Tokyo, Japan) at 37 °C under humidified conditions with 5% CO_2_. DMEM contained 10% fetal bovine serum (FBS; S1810, Biowest, Nuaille, France) and 0.1% streptomycin/penicillin (02008/00906, Meiji Seika Kaisha, Tokyo, Japan). In all experiments, EATCs were pre-incubated at a density of 400 cells/φ 91-mm glass petri dish (FS-90 A, FLAT Co.,Ltd, Chiba, Japan) in 10 ml of culture medium for 48 h. Subsequently, EATCs were adjusted to a concentration of 1.0 × 10⁶ cells/mL and treated with or without EMC (M1204, Tokyo Kasei Kogyo Co., Tokyo, Japan) and palmitic acid (PA; 165 − 00102, FUJIFILM Wako Pure Chemical, Osaka, Japan). EMC was dissolved in dimethyl sulfoxide (048-21985, FUJIFILM Wako Pure Chemical). PA was dissolved in a 10% bovine serum albumin solution free of fatty acids in phosphate-buffered saline (PBS), heated at 37 °C for 30 min, and subsequently underwent sterilizing filtration (0.22 μm).

### Extraction of protein and measurement of protein levels

After the pre-incubation described above, EATCs at a concentration of 1 × 10⁶ cells/ml were seeded at 2 × 10⁶ cells/φ 48-mm glass petri dish (P-2, FLAT Co.,Ltd) or 3.2 × 10⁶ cells /φ 64-mm glass petri dish (FS-60, FLAT Co.,Ltd). EATCs were rinsed twice with PBS and lysed in radioimmunoprecipitation (RIPA) buffer containing 20 µM leupeptin, 1 mM phenylmethylsulfonyl fluoride, and 15 µM pepstatin as protease inhibitors, as well as 5 mM NaF and 1 mM Na₃VO₄ as phosphatase inhibitors. The cell lysates were incubated on ice for 30 min, and intracellular proteins were extracted by freeze-thawing with liquid nitrogen. The total protein concentration in the extract was determined using a Pierce BCA Protein Assay Kit (23227, Thermo Fisher Scientific, Waltham, MA, USA) according to the manufacturer’s protocol. Briefly, 25 µl of the sample was mixed with 200 µl of working reagent and incubated at 37 °C for 30 min. After incubation, the absorbance was measured at 550 nm using a microplate reader (Wallac ARVO SX 1420 multilabel counter; PerkinElmer, Waltham, MA, USA).

### Measurement of glucose consumption

Following the pre-incubation described above, EATCs at a concentration of 1 × 10⁶ cells/ml were seeded at 2 × 10⁶ cells/φ 48-mm glass petri dish. After cultivating EATCs with or without EMC, the culture media were collected. Glucose consumption in the medium was measured using the Glucose Assay Kit-WST (G264, Dojindo, Kumamoto, Japan) following the manufacturer’s protocol. Absorbance at 450 nm, reflecting glucose consumption, was determined using a Wallac ARVO SX 1420 multilabel counter. Glucose consumption was calculated by subtracting the post-culturing glucose concentration from the pre-culturing glucose concentration. Glucose consumption between groups was normalized to intracellular total protein levels.

### Measurement of lactate release levels

EATCs at a concentration of 1 × 10⁶ cells/ml were seeded at 2 × 10⁶ cells/φ 48-mm glass petri dish subsequent to the pre-incubation described above. After cultivating EATCs with or without EMC, the culture media were collected. Lactate release levels in the medium were measured using the Lactate Assay Kit-WST (L256, Dojindo) following the manufacturer’s protocol. Absorbance at 450 nm, reflecting lactate release levels, was determined using a Wallac ARVO SX 1420 multilabel counter. Lactate release levels between groups were normalized to intracellular total protein levels.

### Measurement of ATP levels

After the pre-incubation described above, EATCs at a concentration of 1 × 10⁶ cells/ml were seeded at 2 × 10⁶ cells/φ 48-mm glass petri dish. ATP levels were measured using the CellTiter-Glo 2.0 Assay (Promega, Madison, WI, USA) following the manufacturer’s protocol. Briefly, after culturing EATCs with or without EMC and PA, the cells were collected. The cells suspension was mixed with an equal volume of CellTiter-Glo 2.0 Reagent in a 96-well white plate (204012, Porvair Sciences, Norfolk, UK). After incubating at room temperature for 10 min, luminescence was measured using the Infinite F200 PRO microplate reader (Tecan Group Ltd., Männedorf, Switzerland). ATP levels were normalized to intracellular total protein levels between groups.

### RNA extraction, synthesis of cDNA and quantitative reverse transcription PCR (qRT-PCR)

Following the pre-incubation described above, EATCs at a concentration of 1 × 10⁶ cells/ml were seeded at 2 × 10⁶ cells/φ 48-mm glass petri dish. EATCs were processed for total RNA extraction using the FastGene RNA Basic Kit (FG-80050, Nippon Genetics, Tokyo, Japan) following the manufacturer’s protocol. The quality and quantity of the extracted RNA were assessed using an Agilent 2100 Bioanalyzer (Agilent Technologies, Santa Clara, CA, USA). cDNA was synthesized by reverse transcription of RNA using the ReverTra Ace qPCR RT Master Mix with gDNA Remover (FSQ-301, Toyobo, Osaka, Japan). qRT-PCR analysis was carried out using the GeneAce SYBR qPCR Mix α Low ROX (316–07693, Nippon gene, Tokyo, Japan) and a Stratagene Mx3005P instrument (Agilent Technologies). The *β-actin* gene was used as a reference gene to normalize mRNA expression levels. The mRNA levels were determined using relative quantification (2^−ΔΔCt^). The Primer-BLAST tool (http://www.ncbi.nlm.nih.gov/tools/primer-blast/) was utilized to design all primers (Table 1). The accuracy of the qRT-PCR products was verified through melting curve analysis.

### Measurement of triglyceride (TG) levels

EATCs at a concentration of 1 × 10⁶ cells/ml were seeded at 2 × 10⁶ cells/φ 48-mm glass petri dish subsequent to the pre-incubation described above. After EATCs were rinsed twice with PBS, they were lysed in RIPA buffer and kept on ice for 30 min. Cell lysates were subjected to freeze-thaw cycles in liquid nitrogen, followed by centrifugation to extract intracellular TG. Intracellular TG levels were measured using the LabAssay Triglyceride (632-50991, FUJIFILM Wako Pure Chemical) following the manufacturer’s protocol. Absorbance at 600 nm, reflecting TG levels, was determined using a Wallac ARVO SX 1420 multilabel counter. Intracellular TG levels in each group were normalized to intracellular protein.

### Measurement of DNA content

After the pre-incubation described above, EATCs at a concentration of 1 × 10⁶ cells/ml were seeded at 2 × 10⁶ cells/φ 48-mm glass petri dish. The DNA content of EATCs was quantified using diphenylamine, as previously described^39^. EATCs were rinsed twice with PBS and lysed in 300 µl of 0.4 N perchloric acid. After centrifugation, the precipitate was resuspended in 700 µl of 0.4 N perchloric acid and heated at 70 °C for 20 min. The supernatant was diluted 4-fold with 0.4 N perchloric acid, and 0.4 ml of the diluted sample was mixed with 0.1 ml of ultrapure water and 1 ml of the DNA reaction solution. The DNA reaction solution consisted of 980.3 µl of 88.6 mM diphenylamine in acetic acid, 14.7 µl of sulfuric acid, and 5 µl of 405.3 mM acetaldehyde in ultrapure water. The mixture was incubated overnight at 36 °C, and absorbance was measured at 600 nm using a microplate reader.

### Trypan blue assay

Following the pre-incubation described above, EATCs at a concentration of 1 × 10⁶ cells/mL were seeded at 1 × 10⁵ cells per well in a 96-well microplate and incubated for 24 h with or without EMC and PA. After cultivation, an equal volume of 0.4% trypan blue solution was added to the cell suspension. The numbers of viable and non-viable cells were then counted using a hemocytometer and a microscope. Cell viability was calculated using the following formula: cell viability (%) = [(total cells – non-viable cells) / total cells] × 100.

### Western blot analysis

After the pre-incubation, EATCs at a concentration of 1 × 10⁶ cells/ml were seeded at 3.2 × 10⁶ cells /φ 58-mm glass petri dish. Protein extraction and quantification were performed as described above. SDS-PAGE was employed to separate proteins using an AE-8155 myPowerII 500 (ATTO, Tokyo, Japan), and the proteins were subsequently transferred to a PVDF membrane (IPFL00010; Merck Millipore, Billerica, MA, USA). The membranes were blocked at room temperature for 1 h using skim milk dissolved in Tris-buffered saline containing Tween 20. Subsequently, the primary antibody incubation was performed at room temperature for 2 h. The primary antibodies used were anti-SREBP1 (sc-13551, 1:600, Santa Cruz Biotechnology, Santa Cruz, CA, USA), anti-c-Myc (sc-40, 1:1000, Santa Cruz Biotechnology), anti-phospho-c-Myc (Ser62) (#13748, 1:1000, Cell Signaling Technology, Danvers, MA, USA), and anti-GAPDH (#5174, 1:15000, Cell Signaling Technology). Membranes were incubated with Goat anti-Mouse IgG (H + L) Secondary Antibody, Biotin (#31800, 1:2000, Thermo Fisher Scientific) or Anti-rabbit IgG (H + L), Biotinylated Antibody (#14708, 1:2000, Cell Signaling Technology) at room temperature for 1 h, followed by exposure to horseradish peroxidase-conjugated streptavidin (P0397, Agilent Technologies) for an additional hour. Chemiluminescent bands of the proteins were detected using EzWestLumi Plus (2332638, ATTO) and quantified with ImageJ.

### Statistical analysis

All numerical data are expressed as mean ± standard deviation (SD). Comparisons among the groups were performed by one-way ANOVA, followed by the Tukey-Kramer test in Statcel-4 (OMS Inc., Tokorozawa, Japan). Comparisons between two groups were evaluated for statistical significance by Student’s t-test. Differences were considered statistically significant at *p* < 0.05.

**Table 1:** Primer sequences.

Gene	Primer sequences (5’ → 3’)	Tm (℃)	GeneBank No.
*Acly*	*CCTTTGACAGCGGCATCATT*	63.4	NM_001199296.1
	TTCAGGATAAGATTTGGCTTCTTGG	63.8	
*Acc1*	*CTGCAGAAACTCATCCTCTCGG*	63.6	NM_133360.3
	GCGGTGTTGTACGCTGTTGA	63.2	
*Fasn*	*GAATACACAGCCACCGACCG*	63.8	NM_007988.3
	CACCAGAAGGTCAAGGGCAC	63.0	
*Cpt1a*	*GGCTATGGTCAAGGTCTTCTCG*	62.4	NM_013495.2
	GTGCTGTCATGCGTTGGAAGT	63.6	
*Cpt1b*	*CAGGCAAAGAGACAGACTTGCTA*	61.1	NM_009948.2
	GCCCTCATAGAGCCAGACCTT	62.0	
*SREBP1*	*GAGCTGCGTGGTTTCCAACA*	64.6	NM_011480.4
	GTGGCCTCATGTAGGAATACCCTC	64.1	
*b-actin*	*CATCCGTAAAGACCTCTATGCCAAC*	64.1	NM_007393.5
	ATGGAGCCACCGATCCACA	64.9	

*Acly*: ATP citrate lyase, *Acc1*: acetyl-CoA carboxylase alpha, *Fasn*: fatty acid synthase, *Cpt1a*: carnitine palmitoyltransferase 1a, *Cpt1b*: *carnitine palmitoyltransferase 1b*, *SREBP1*: sterol regulatory element binding transcription factor 1, and *β-Actin*: Beta-actin.

## Electronic supplementary material

Below is the link to the electronic supplementary material.

Supplementary Material 1

## Data Availability

The datasets used and/or analyzed during the current study available from the corresponding author on reasonable request.
