# Supplementary material for: Ethyl p-methoxycinnamate inhibits tumor growth by suppressing of fatty acid synthesis and depleting ATP
Source: Sci Rep. 2025 May 2;15:15317. doi: 10.1038/s41598-025-00131-1 (PMC12046015; doi:10.1038/s41598-025-00131-1)
Supplement: Supplementary file 1 — Supplementary Material 1 [file 41598_2025_131_MOESM1_ESM.pdf]

Fig. S1. Non-adjusted full images of immunoblots in Fig. 16. Images were obtained using AE-9300 Ez-Capture MG (ATTO).

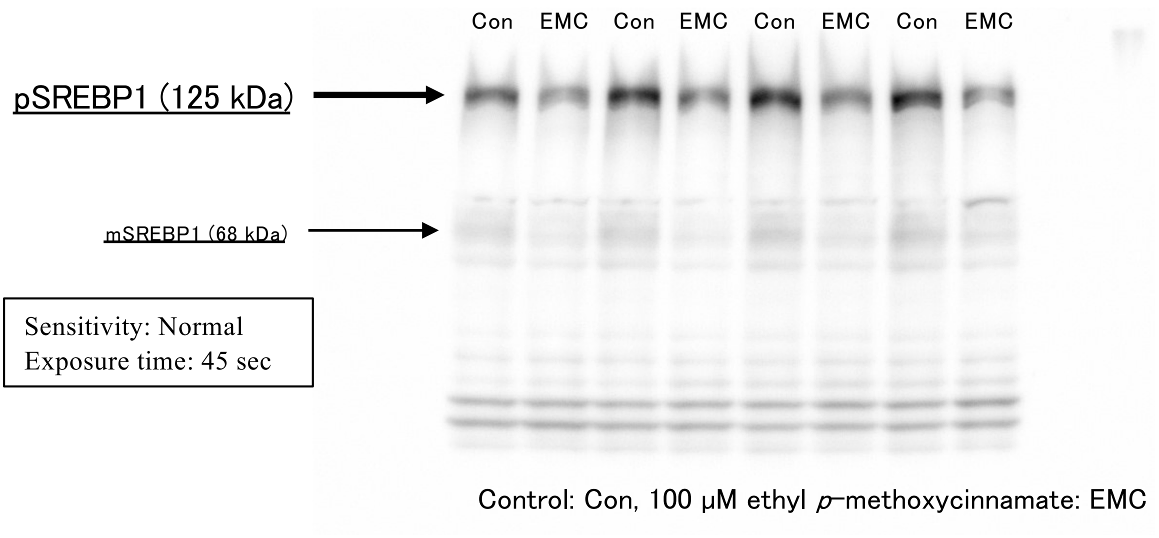

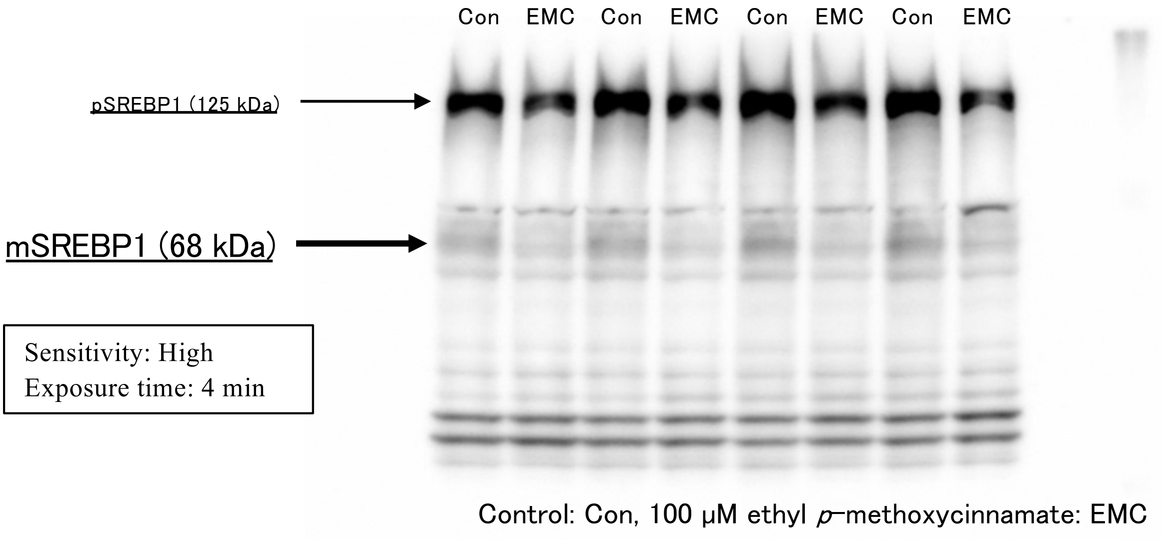

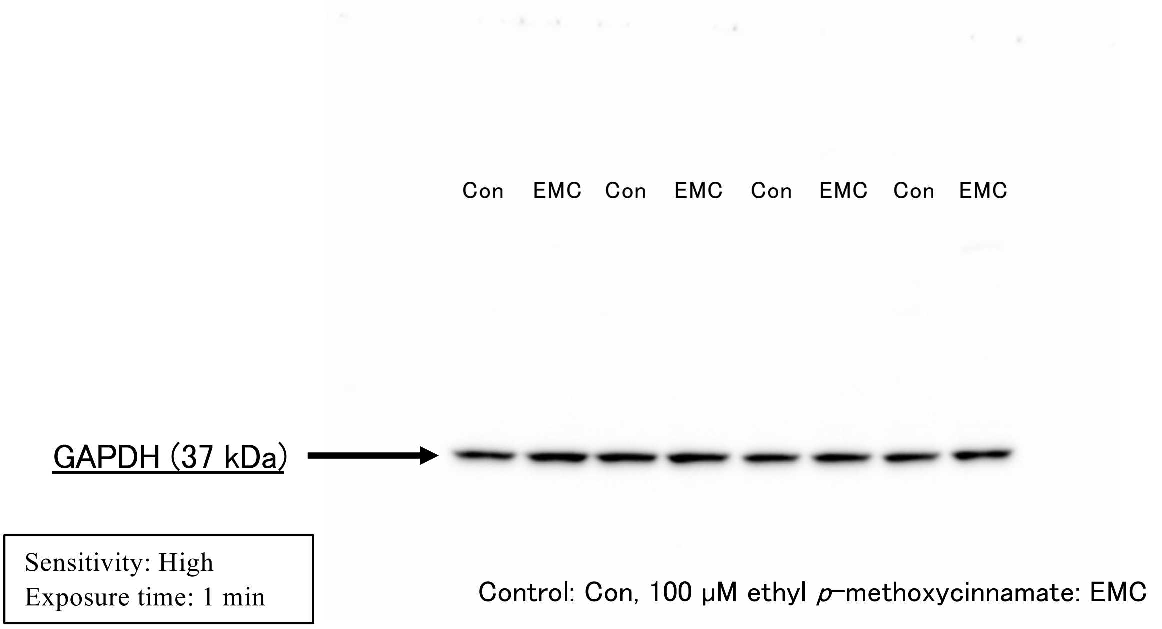

Fig. S2. Non-adjusted full images of immunoblots in Fig. 17. Images were obtained using AE-9300 Ez-Capture MG (ATTO).

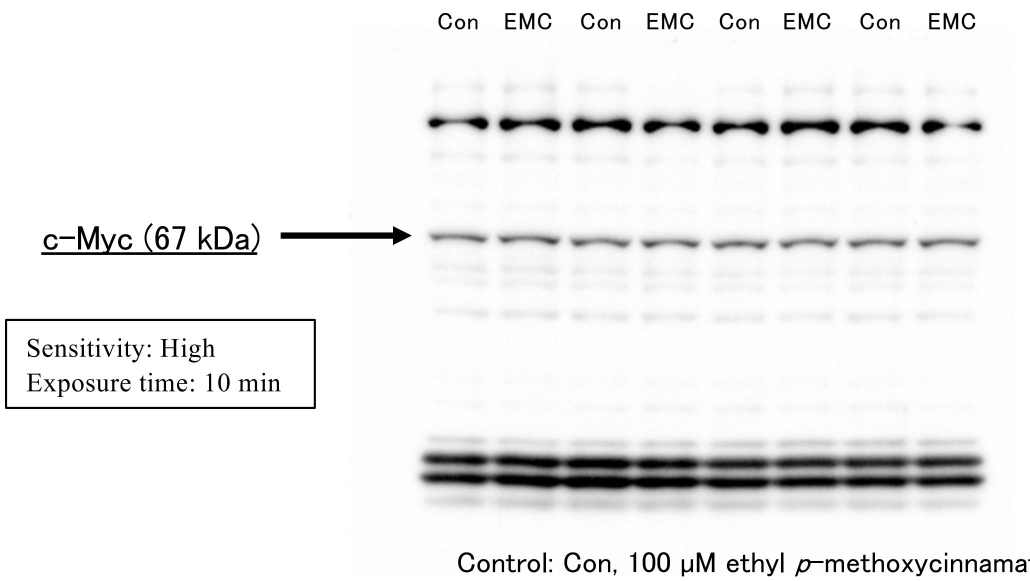

Control: Con, 100 µM ethyl *p*-methoxycinnamate: EMC

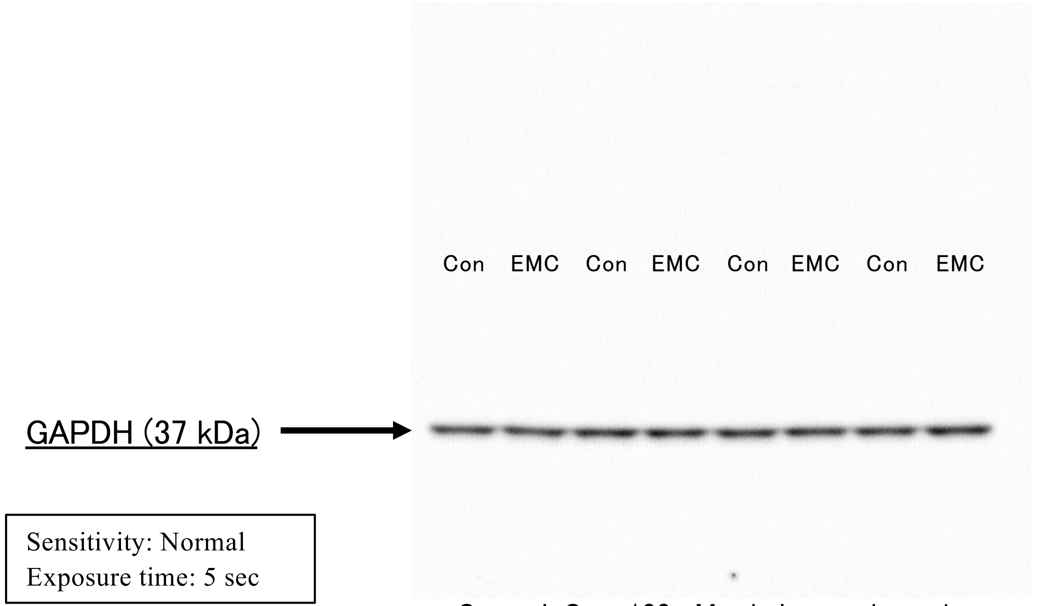

Control: Con, 100 µM ethyl *p*-methoxycinnamate: EMC

Fig. S3. Non-adjusted full images of immunoblots in Fig. 18. Images were obtained using AE-9300 Ez-Capture MG (ATTO).

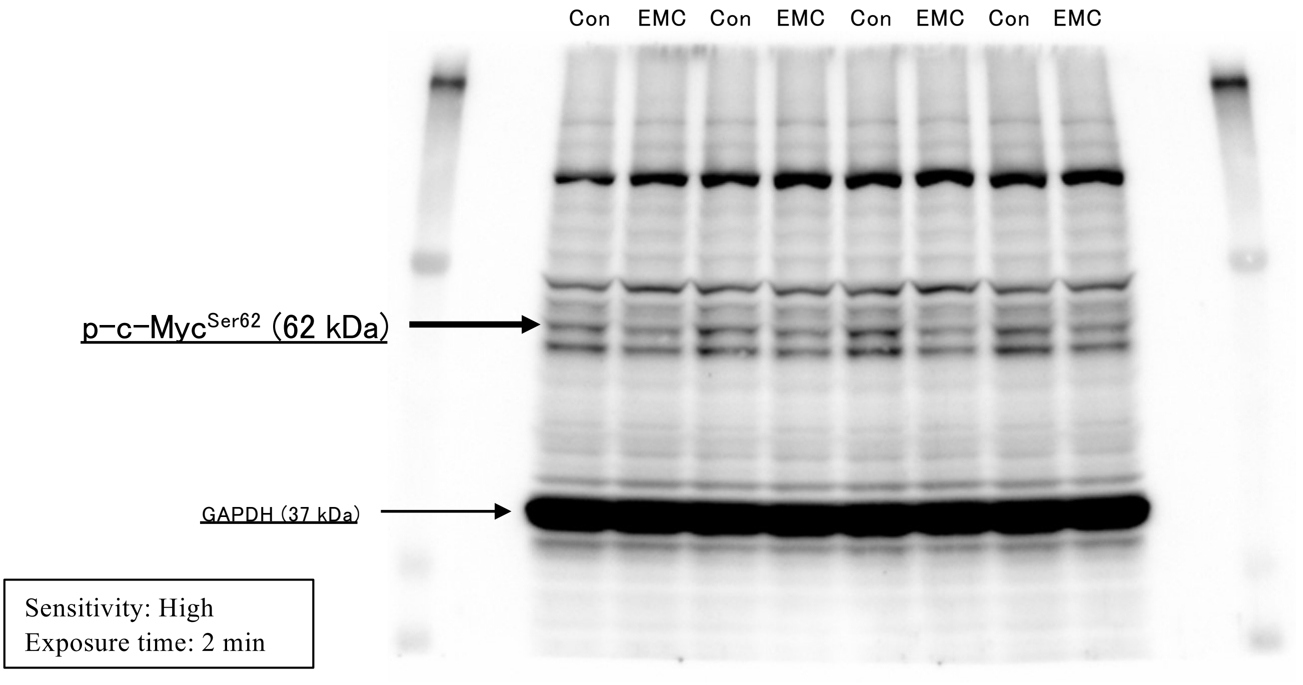

Control: Con, 100 μM ethyl *p*-methoxycinnamate: EMC

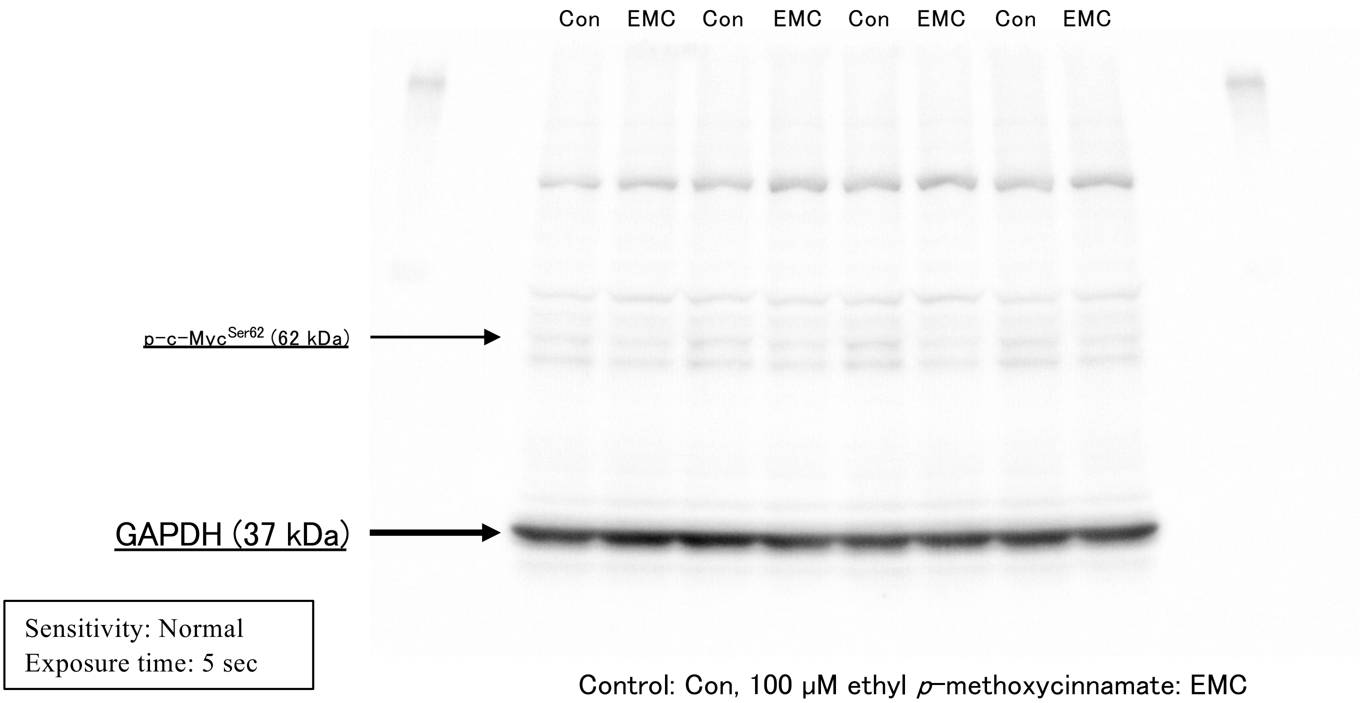

Control: Con, 100 μM ethyl *p*-methoxycinnamate: EMC
